# A Rasch model analysis of two interpretations of ‘not relevant’ responses on the Dermatology Life Quality Index (DLQI)

**DOI:** 10.1007/s11136-021-02803-7

**Published:** 2021-03-08

**Authors:** Fanni Rencz, Ariel Z. Mitev, Ákos Szabó, Zsuzsanna Beretzky, Adrienn K. Poór, Péter Holló, Norbert Wikonkál, Miklós Sárdy, Sarolta Kárpáti, Andrea Szegedi, Éva Remenyik, Valentin Brodszky

**Affiliations:** 1 Department of Health Economics, Corvinus University of Budapest, 8 Fővám tér, 1093 Budapest, Hungary; 2 Hungarian Academy of Sciences, Premium Postdoctoral Research Programme, 7 Nádor u, 1051 Budapest, Hungary; 3 Institute of Marketing, Corvinus University of Budapest, 8 Fővám tér, 1093 Budapest, Hungary; 4 Doctoral School of Clinical Medicine, Semmelweis University, 26 Üllői út, 1085 Budapest, Hungary; 5 Doctoral School of Business and Management, Corvinus University of Budapest, 8 Fővám tér, 1093 Budapest, Hungary; 6 Department of Dermatology, Venereology and Dermatooncology, Faculty of Medicine, Semmelweis University, 41 Mária u, 1085 Budapest, Hungary; 7 Departments of Dermatology, Faculty of Medicine, University of Debrecen, 98 Nagyerdei krt, 4032 Debrecen, Hungary; 8 Department of Dermatological Allergology, Faculty of Medicine, University of Debrecen, 98 Nagyerdei krt, 4032 Debrecen, Hungary

**Keywords:** Psoriasis, Dermatology Life Quality Index, Psychometrics, Rasch model, ‘not relevant’ response, DLQI-R

## Abstract

**Purpose:**

Eight of the ten items of the Dermatology Life Quality Index (DLQI) have a ‘not relevant’ response (NRR) option. There are two possible ways to interpret NRRs: they may be considered ‘not at all’ or missing responses. We aim to compare the measurement performance of the DLQI in psoriasis patients when NRRs are scored as ‘0’ (hereafter zero-scoring) and ‘missing’ (hereafter missing-scoring) using Rasch model analysis.

**Methods:**

Data of 425 patients with psoriasis from two earlier cross-sectional surveys were re-analysed. All patients completed the paper-based Hungarian version of the DLQI. A partial credit model was applied. The following model assumptions and measurement properties were tested: dimensionality, item fit, person reliability, order of response options and differential item functioning (DIF).

**Results:**

Principal component analysis of the residuals of the Rasch model confirmed the unidimensional structure of the DLQI. Person separation reliability indices were similar with zero-scoring (0.910) and missing-scoring (0.914) NRRs. With zero-scoring, items 6 (sport), 7 (working/studying) and 9 (sexual difficulties) suffered from item misfit and item-level disordering. With missing-scoring, no misfit was observed and only item 7 was illogically ordered. Six and three items showed DIF for gender and age, respectively, that were reduced to four and three by missing-scoring.

**Conclusions:**

Missing-scoring NRRs resulted in an improved measurement performance of the scale. DLQI scores of patients with at least one vs. no NRRs cannot be directly compared. Our findings provide further empirical support to the DLQI-R scoring modification that treats NRRs as missing and replaces them with the average score of the relevant items.

## Introduction

The Dermatology Life Quality Index (DLQI) is the most frequent health-related quality of life (HRQoL) measure in patients with psoriasis, used in a range of settings, including consultations, clinical trials as well as for treatment decisions [1, 2]. It is the most commonly used HRQoL instrument in randomised controlled trials for psoriasis [3]. In clinical guidelines, DLQI along with Psoriasis Area and Severity Index (PASI) is considered a useful benchmark to define moderate-to-severe psoriasis, to set out eligibility criteria for systemic treatments, including biologics, and to evaluate the effectiveness of treatments [4–6]. The DLQI has been translated to over 100 languages, and a recent study found 40 some countries using the DLQI in their national psoriasis treatment guidelines and/or registries [7].

In spite of the nearly three decades of experience accumulated with the DLQI, the dermatological community has just recently started to study the matter of ‘not relevant’ responses (NRRs) on the DLQI. Studies from the US and Europe reported that one or more items of the DLQI are irrelevant for 22.1–48.0% of psoriasis patients [8]. Prior work suggests that female gender, lower education level, single, widowed or divorced marital status and unemployed or disabled employment status were associated with increased odds of having at least one NRR [8–11]. It has also been described that psoriasis patients who responded ‘not relevant’ had more severe disease than those who responded ‘not at all’ in questionnaire items [9].

**Fig.1:**
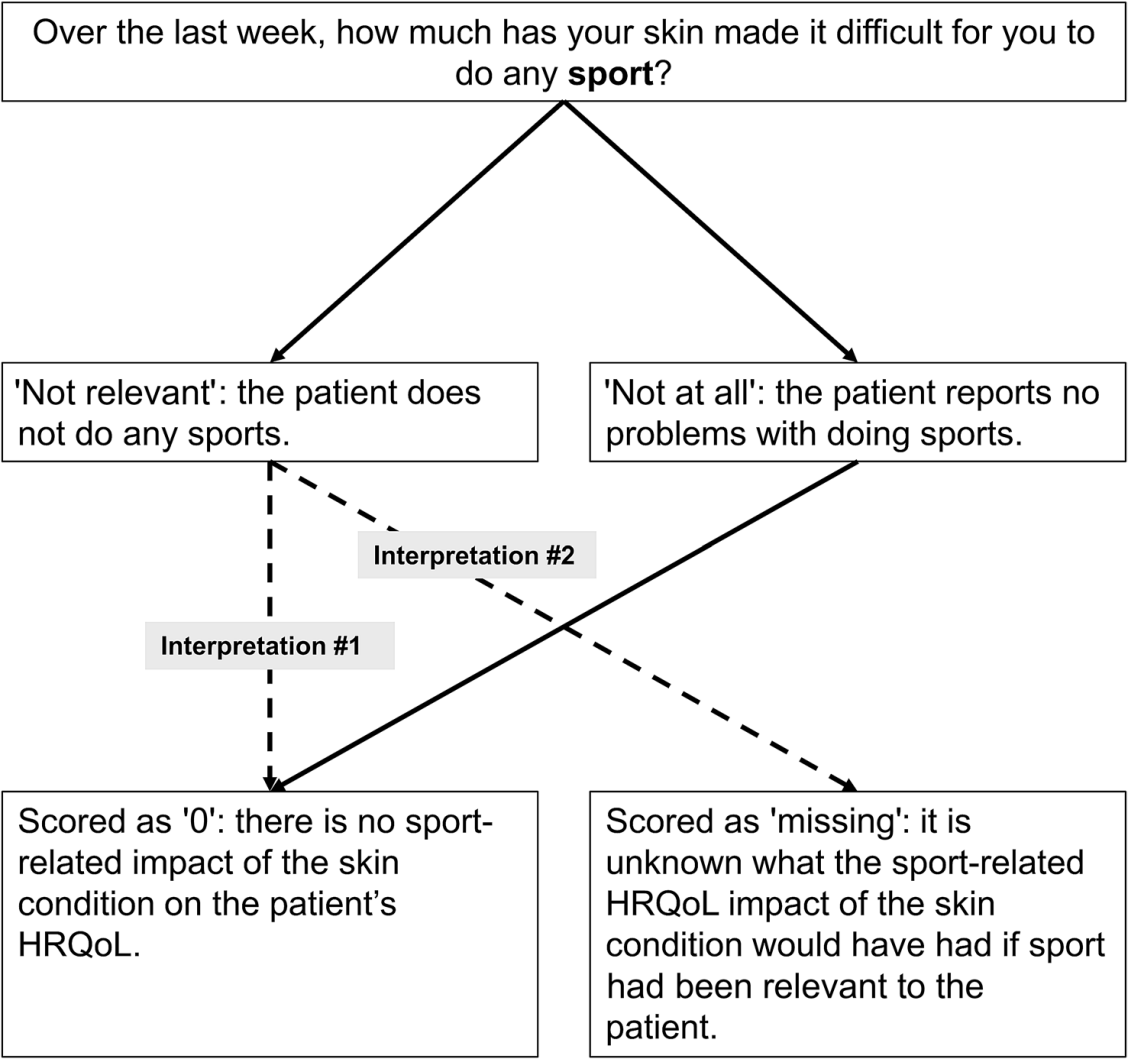
The two possible interpretations of ‘not relevant’ responses on the DLQI. *DLQI* Dermatology Life Quality Index, *HRQoL* health-related quality of life

NRRs appear in eight of the 10 items on the DLQI. These items are related to the following areas of HRQoL: shopping/home/gardening, clothing, social/leisure activities, sport, working/studying, interpersonal problems, sexual difficulties and treatment difficulties. There are two possibilities to interpret NRRs (Fig. 1). Let us take the example of a patient that is not practicing any sports, therefore responds ‘not relevant’ to item 6 (sport) on the DLQI. According to the first interpretation of the NRR – that is also in line with the official scoring of the DLQI – there is no sport-related impact of the skin condition on this patient’s HRQoL that should be considered equivalent to a ‘not at all’ response and scored as ‘0′ (hereafter referred to as zero-scoring). The second interpretation is to consider that as sport is not relevant to the patient it is unknown what the sport-related HRQoL impact of the skin condition would have had if sport had been relevant to this patient. In this second approach, the response does not provide any information on the measured concept, and thus, is considered as a missing response (hereafter referred to as missing-scoring). This second interpretation of the NRR is well aligned with the DLQI-Relevant (DLQI-R) scoring modification of the DLQI that treats NRRs as missing and replaces them with the average score of the relevant items [12].

The two parallel existing interpretations of the NRR option may compromise the comparability of responses and DLQI scores across patients. DLQI scores obtained with one or several NRRs may not be compared to the DLQI scores obtained on a full set of ‘relevant’ responses. Moreover, if two different items are not relevant to two different patients, it is unclear if comparability of their scores may be ensured.

All previous studies focusing on NRRs applied classical test theory as a framework to guide validation [9–11, 13]. An alternative measurement approach, a Rasch model analysis, may offer numerous advantages over classical test theory in evaluating psychometric performance of scales [14]. So far, a small number of studies have applied Rasch models to examine the psychometric properties of the DLQI in various skin diseases [15–21]; however, only one study concerned with psoriasis alone, and no studies differentiated between the two possible interpretations of NRRs. The objective of this study is therefore to (i) evaluate the psychometric properties of the DLQI in patients with psoriasis using a Rasch model analysis and (ii) to compare the measurement performance of DLQI when NRRs are scored as ‘0′ and ‘missing’.

## Methods

### Study design and patients

Data from two cross-sectional questionnaire surveys among psoriasis patients were pooled for this study. These surveys have been undertaken at two university dermatology clinics in Hungary. Eligible patients were 18 years or older, diagnosed with psoriasis by a dermatologist and able to understand and complete the questionnaire. In the first survey, patients had to meet further eligibility criteria, including having moderate-to-severe psoriasis or having been treated with systemic non-biological or biological therapy for at least 12 months before the survey [22]. Diagnosis of moderate-to-severe psoriasis was established based on the definition of the European consensus: [body surface area > 10 or Psoriasis Area and Severity Index (PASI) > 10] and DLQI > 10 [4]. The second survey recruited both in- and outpatients regardless of disease severity [23]. There were no exclusion criteria other than failure to meet the inclusion criteria.

The dataset included patients’ DLQI responses, PASI score and the following demographic and clinical characteristics: age, gender, disease duration, clinical subtype and treatment at the time of the survey.

### Dermatology Life Quality Index (DLQI)

DLQI is intended to assess the HRQoL impairment of adult patients (aged ≥ 16 years) on the preceding week. It has 10 items covering the following six aspects of HRQoL: symptoms and feelings, daily activities, leisure, work/school, personal relationships, and treatment. Each item is answered on a 4-point scale scored as follows: ‘not at all’ = 0, ‘a little’ = 1, ‘a lot’ = 2 and ‘very much’ = 3. NRR options are available for items 3–10. Item scores are added up to give a minimum score of 0 and maximum score of 30, where a higher DLQI total score indicates a greater degree of HRQoL impairment. The Hungarian version of the paper-based DLQI was used in the surveys.

### Rasch model

The Rasch model is a mathematical model that describes the relationship between individuals’ ‘latent trait’ (i.e. impairment in HRQoL) and how they respond to items on a scale. A polytomous Rasch model (partial credit model) was applied to analyse the psychometric properties of the DLQI [24]. First, we used a likelihood-ratio test to determine whether the rating scale or the partial credit model with conditional likelihood estimation was most appropriate [25]. We tested the following key assumptions and properties of the Rasch model: dimensionality, item fit, person reliability, order of response options and item invariance. A person-item map was depicted to place both persons and items on the same interval-level scale, so that they can be compared [26]. Two separate analyses were carried out with zero-scoring and missing-scoring NRRs, respectively. By missing-scoring each NRR, we took advantage of the ability of the Rasch model to handle missing responses by simply not including that item for that patient in the estimation.

#### Dimensionality

To analyse dimensionality, we performed a principal component analysis (PCA) using orthogonal varimax rotation on the standardized residuals of the Rasch model. Residuals were defined as the discrepancy between the observed and predicted values of the model. The DLQI was considered unidimensional if all 10 items underlined the same latent trait. A possible presence of additional dimensions was considered when the eigenvalues of the residual components were ≥ 2 [27]. Response dependency was evaluated via the correlation between the items’ standardized residuals, whereby correlation coefficients of 0.3 and above were considered unacceptable [28].

#### Person separation reliability

In order to determine the internal reliability of the DLQI in differentiating between patients with different level of HRQoL impairment, we computed person separation reliability. Values range from 0 to 1, where a separation reliability value of > 0.8 indicates an acceptable reliability [27].

#### Item fit

The fit of the data to the Rasch model was investigated with reference to χ^2^-fit [29] and infit and outfit unstandardized mean square (MNSQ) statistics. A significant χ^2^-fit statistic indicates misfit to the model. The infit and outfit MNSQ values range between zero and positive infinity, where 1 indicates a perfect fit of data to the Rasch model. Infit and outfit MNSQ values ranging between 0.5 and 1.5 are considered indicative of a well-fitting model [30, 31]. A lower value suggests overfit between the items and the model (i.e. items are too discriminating) and a higher value indicates underfit (i.e. unpredictability of data).

#### Order of response options

Response options of DLQI items (scored from 0 to 3) are expected to follow each other in a monotonic order; thus, ranging from the least severe to the most severe. In other words, the more problems with HRQoL patients have in a certain item, the higher their probability of endorsing it is. This relationship between HRQoL impairment of patients and their responses on DLQI items was depicted by item characteristic curves (ICCs).

#### Item invariance

A lack of item invariance [i.e. differential item functioning (DIF)] means that different subgroups respond differently to certain DLQI items, after controlling for differences in patients’ overall HRQoL [32]. Two types of DIF can be identified: uniform DIF that is constant across ability levels and non-uniform DIF that varies across ability levels. The presence of DIF was assessed across gender (female or male) and age [below or above median age (49 years)] by applying a likelihood-ratio test [33, 34].

For all analyses a p < 0.05 was considered statistically significant. The Rasch model analysis was undertaken using the *eRM* package in R version 3.6.1 (Vienna, Austria) [35, 36] and differential item functioning was tested using the *DIFLRT* macro [37] in IBM SPSS Statistics for Windows, version 25.0 (IBM Corp., Armonk, N.Y., USA).

## Results

### Patient population

Overall, 436 patients with psoriasis participated in the two surveys. Data of 11 patients with missing responses on one or more DLQI items were excluded, and as a result, the final sample consisted of 425 patients. Nearly two-thirds of the patients (64.9%) were male (Table 1). Patients’ age ranged between 18 and 86 years, with a mean of 49.2 ± 14.3 years. Mean disease duration was 19.8 ± 12.2 years. The most common clinical subtypes were chronic plaque psoriasis (73.4%), scalp psoriasis (48.2%) and nail psoriasis (45.6%). Over one-third of the patients were diagnosed with psoriatic arthritis (35.8%). The proportion of patients with a PASI score ≥ 10 was 66.4%.

**Table 1:** Characteristics of the psoriasis patient population (n = 425)

Variables	Mean (SD) or n (%)
Age (years)	49.2 (14.3)
Disease duration (years)	19.8 (12.2)
Gender	
Female	149 (35.1%)
Male	276 (64.9%)
Clinical subtype^*a*^	
Chronic plaque psoriasis	312 (73.4%)
Erythrodermic	7 (1.6%)
Facial and/or inverse	78 (18.4%)
Guttate	27 (6.4%)
Nail	194 (45.6%)
Palmoplantar	29 (6.8%)
Psoriatic arthritis	152 (35.8%)
Pustular	2 (0.5%)
Scalp	205 (48.2%)
Treatments	
None	31 (7.3%)
Topical (only)	103 (24.2%)
Systemic non-biological	107 (25.2%)
Biological	184 (43.3%)
Disease severity (PASI 0–72)	
Mean	8.4 (9.5)
PASI ≤ 10	143 (33.6%)
PASI > 10	282 (66.4%)
DLQI (0–30)^*b*^	
Mean	6.8 (7.4)
0–1	146 (34.4%)
2–5	94 (22.1%)
6–10	71 (16.7%)
11–20	90 (21.2%)
21–30	24 (5.6%)

*DLQI* Dermatology Life Quality Index, *PASI* Psoriasis Area and Severity Index

***a:*** Combinations are possible

***b:*** DLQI scores are categorised according to the Hongbo’s DLQI score bands [56]

### Item descriptives

**Fig.2:**
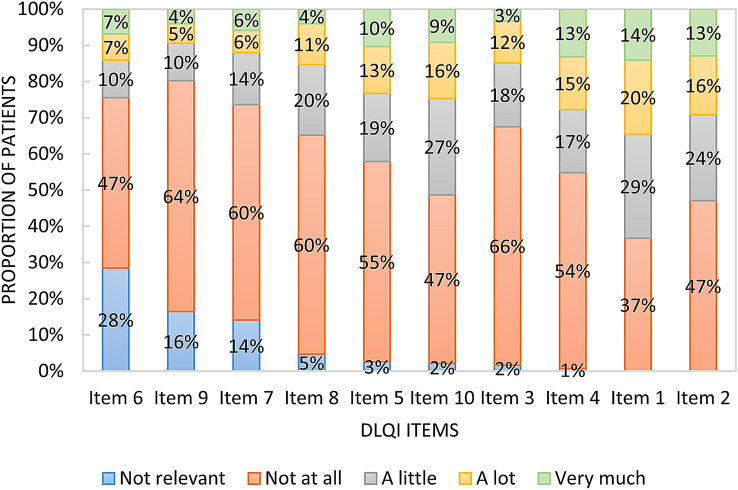
Distribution of responses on the 10 items of the DLQI ordered in relation to their proportion of ‘not relevant’ responses (n = 425). Item 1 = itchy/sore/painful/stinging; Item 2 = embarrassed/self conscious; Item 3 = shopping/home/gardening; Item 4 = clothes; Item 5 = social/leisure activities; Item 6 = sport; Item 7 = working/studying; Item 8 = interpersonal problems; Item 9 = sexual difficulties; Item 10 = treatment difficulties. *DLQI* Dermatology Life Quality Index

Mean DLQI score was 6.8 ± 7.4. Overall, 113 patients (26.6%) had a DLQI score of zero, while two patients (0.5%) achieved the maximum of 30 points. A total of 84 (19.8%) patients had one, 49 (11.5%) had two, 22 (5.2%) had three, 7 (1.6%) had four, and 4 (0.9%) had over four NRRs. Relative frequencies of responses on the 10 items of the DLQI are provided in Fig. 2. The largest proportion of NRRs occurred in items 6 (sport), 9 (sexual difficulties) and 7 (working/studying).

### Dimensionality

PCA on the residuals of the Rasch model revealed one factor explaining 60.9% of the variance in DLQI. All the eigenvalues of the residuals (range 0.160–1.699) of the latent trait were < 2, and the correlations between the items’ standardized residuals (range │0.001│–│0.282│) were below 0.3 supporting the unidimensional construct of the DLQI.

### Person separation reliability

Person separation reliability values for the DLQI were slightly better (0.914) with missing-scoring in comparison with zero-scoring NRRs (0.910).

### Person and item fit

Overall, 3.76% and 2.90% of patients misfitted the Rasch model when NRRs were scored as ‘0′ and missing, respectively. With zero-scoring NRRs, the χ^2^ fit statistic detected three items to misfit to the Rasch model: items 6 (sport) (p < 0.001), 7 (working/studying) (p = 0.034) and item 9 (sexual difficulties) (p = 0.0498) (Table 2). No items misfitted to the model in case of scoring NRRs as missing. With zero-scoring, the outfit and infit MNSQ statistics ranged between 0.564 [item 5 (social/leisure activities)] to 1.378 [item 6 (sport)] and between 0.611 [item 5 (social/leisure activities)] to 1.212 [item 9 (sexual difficulties)], respectively. With missing-scoring, the outfit and infit MNSQ statistics were between 0.561 [item 5 (social/leisure activities)] and 1.131 [item 7 (working/studying)] and 0.598 [item 5 (social/leisure activities)] and 1.291 [item 9 (sexual difficulties)], respectively. These values are within the range of the commonly accepted cut-offs (0.5–1.5).

**Table 2:** Item locations, fit statistics and DIF of DLQI items

Items	Item location (difficulty)	Item fit					Differential item functioning (DIF)	
		χ^2^	df	p-value	MNSQ (outfit)	MNSQ (infit)	DIF	No DIF
NRRs coded as ‘0’								
Item 1 (itchy/sore/painful/stinging)	− 0.871	343.7	309	0.085	1.109	1.098	Age (nU)	Gender
Item 2 (embarrassed/self conscious)	− 0.418	228.8	309	1.000	0.738	0.760	Gender (U)	Age
Item 3 (shopping/home/gardening)	0.882	199.8	309	1.000	0.645	0.717	Age (U) Gender (U)	–
Item 4 (clothes)	− 0.205	290.4	309	0.770	0.937	0.891	Gender (U)	Age
Item 5 (social/leisure activities)	0.049	174.9	309	1.000	0.564	0.611	Gender (U)	Age
Item 6 (sport)	0.736	427.3	309	*** < 0.001***	1.378	1.066	Age (U) Gender (U)	–
Item 7 (working/studying)	0.793	355.9	309	***0.034***	1.148	1.050	–	Age Gender
Item 8 (interpersonal problems)	0.743	248.5	309	0.995	0.802	0.880	–	Age Gender
Item 9 (sexual difficulties)	1.125	351.1	309	***0.0498***	1.132	1.212	Gender (U)	Age
Item 10 (treatment difficulties)	− 0.144	288.9	309	0.788	0.932	0.942	–	Age Gender
NRRs coded as missing								
Item 1 (itchy/sore/painful/stinging)	− 0.743	342.1	309	0.094	1.104	1.098	Age (U)	Gender
Item 2 (embarrassed/self conscious)	− 0.262	234.2	309	0.999	0.755	0.768	Gender (U)	Age
Item 3 (shopping/home/gardening)	1.110	202.9	305	1.000	0.663	0.735	Age (U) Gender (U)	–
Item 4 (clothes)	− 0.038	326.5	307	0.212	1.060	0.975	Gender (U)	Age
Item 5 (social/leisure activities)	0.157	169.0	300	1.000	0.561	0.598	–	Age Gender
Item 6 (sport)	0.412	207.6	214	0.611	0.965	0.949	Gender (U)	Age
Item 7 (working/studying)	0.825	299.8	264	0.064	1.131	1.036	–	Age Gender
Item 8 (interpersonal problems)	0.895	249.6	293	0.969	0.849	0.922	–	Age Gender
Item 9 (sexual difficulties)	1.095	263.9	247	0.220	1.064	1.261	Age (nU)	Gender
Item 10 (treatment difficulties)	0.001	290.2	305	0.720	0.948	0.955	–	Age Gender

Coding of variables: Age: 0 =  < 49 years (median age), 1 =  ≥ 49 years

*DIF* differential item functioning, *DLQI* Dermatology Life Quality Index, *MNSQ* unstandardized mean square statistics, *NRR* ‘not relevant’ response, *nU* non-uniform DIF, *U* uniform DIF

### Order of response options

**Fig.3:**
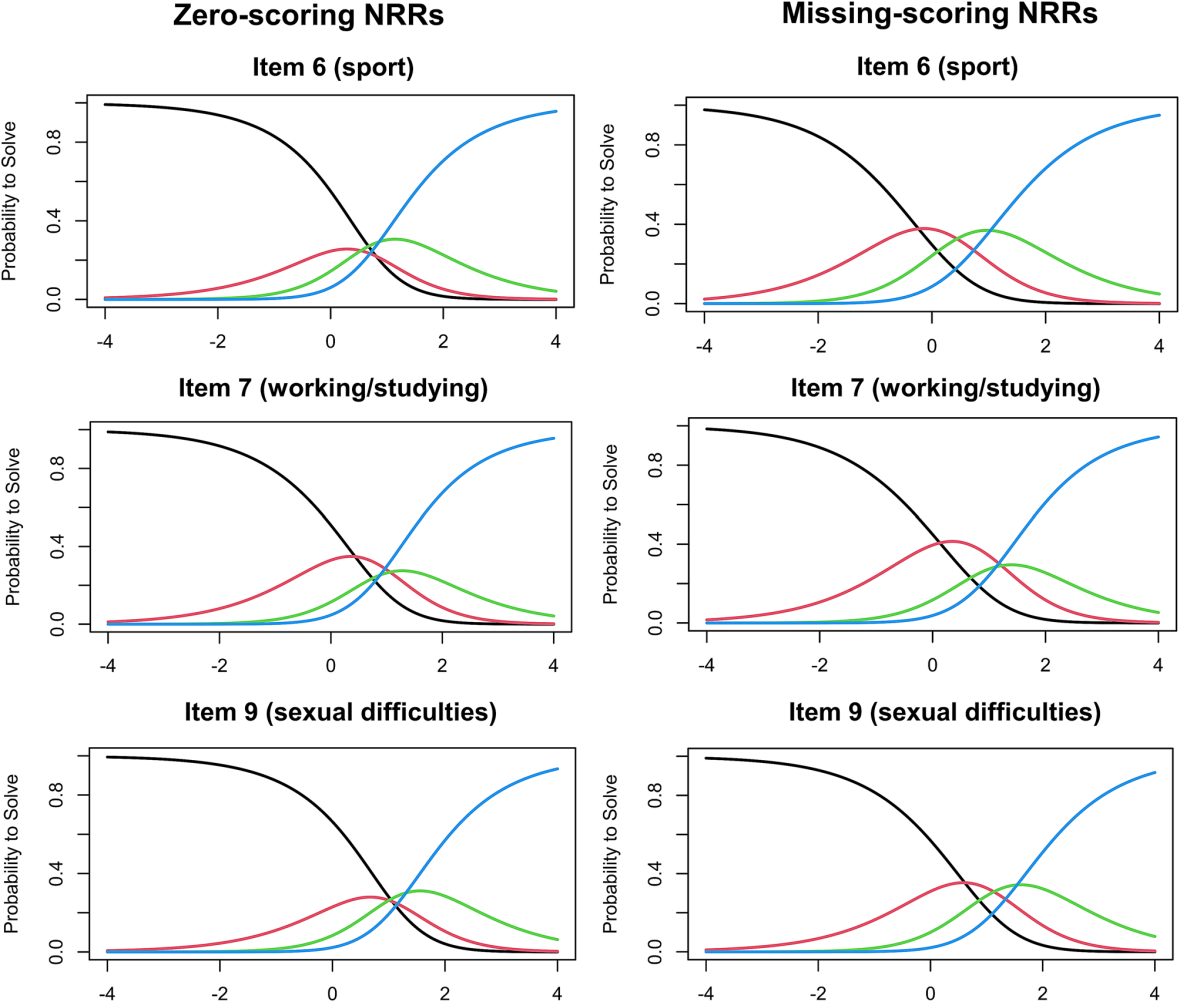
Item characteristic curves (ICC) for DLQI items 6, 7 and 9. The latent trait (i.e. HRQoL impairment) is measured along axis x, while axis y indicates the probability of endorsing an item. The point on axis x at which the curve for an item crosses the 0.5 probability level on axis y serves as an index of item difficulty indicating where 50% of the patients endorse a given item. Items with lower item difficulty values are considered to be ‘easier’ and expected to be endorsed at lower HRQoL impairment [14]. Curves: ‘0 scoring’ NRRs: black: 0 (‘not at all’ or ‘not relevant’), red: 1 (‘a little’), green: 2 (‘a lot’), blue: 3 (‘very much’). ‘Missing scoring’: black: 0 (‘not at all’), red: 1 (‘a little’), green: 2 (‘a lot’), blue: 3 (‘very much’). *DLQI* Dermatology Life Quality Index, *HRQoL* health related quality of life, *NRR* ‘not relevant’ response

When zero-scoring, response thresholds were disordered for items 6 (sport), 7 (working/studying) and 9 (sexual difficulties) (Fig. 3). Response options 1 (‘a little’) and 2 (‘a lot’) did not follow the monotonic order for items 6 (sport) and 9 (sexual difficulties), and 2 (‘a lot’) and 3 (‘very much’) for item 7 (working/studying). Conversely, with missing-scoring NRRs, only response options 2 (‘a lot’) and 3 (‘very much’) were illogically ordered for item 7 (working/studying).

### Person-item map

**Fig.4:**
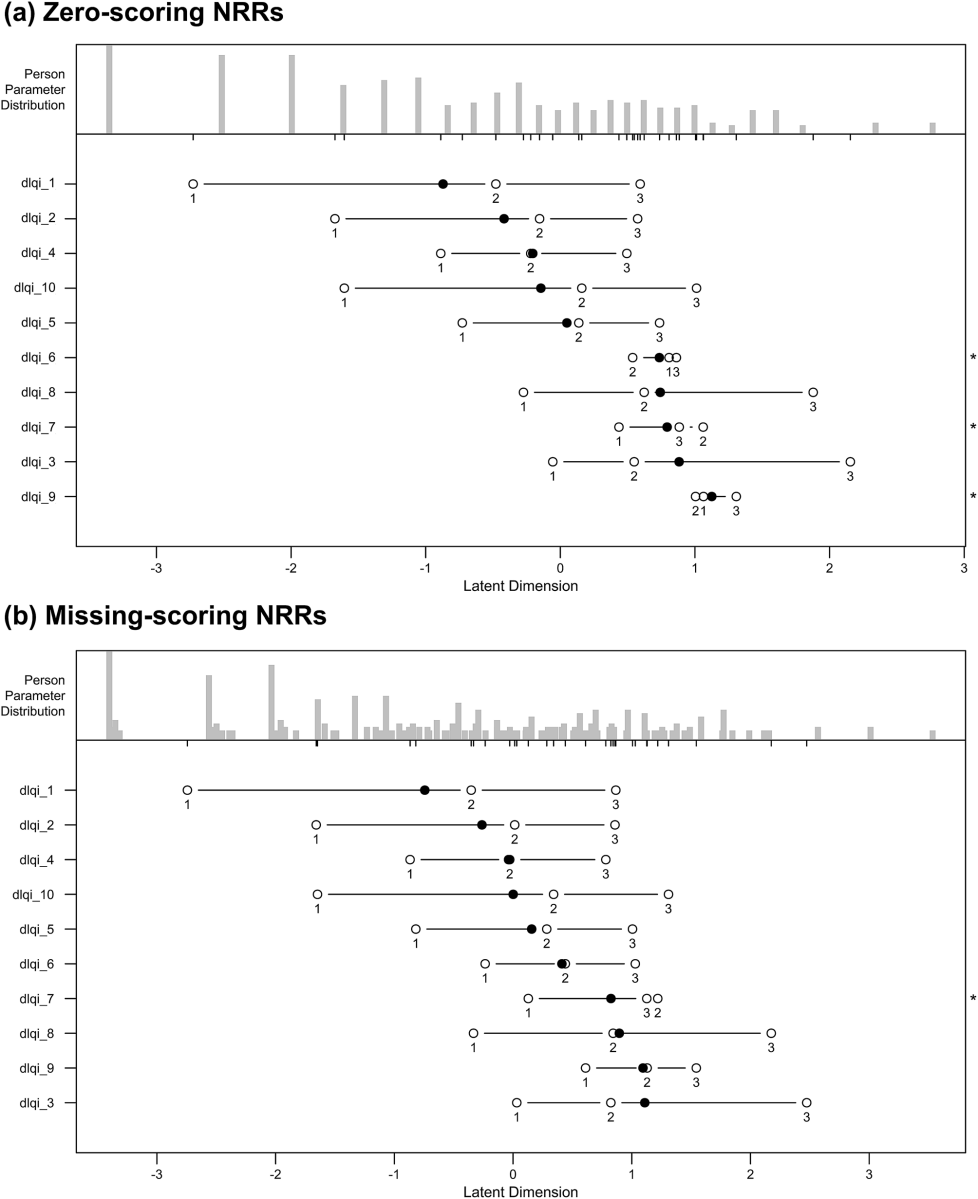
Person-item maps. Each map has two panels, the upper panel shows the histogram of patients’ HRQoL impairment estimates, while the lower panel indicates the locations of items ordered in relation to their difficulty using a logit scale. The items that are the easiest to endorse are positioned on the left and the most difficult (i.e. require more impairment in HRQoL to endorse) items are located in the right. Items should ideally be located along the whole scale to meaningfully measure the HRQoL impairment across the entire spectrum of patients. dlqi_1 = itchy/sore/painful/stinging; dlqi_2 = embarrassed/self conscious; dlqi_3 = shopping/home/gardening; dlqi_4 = clothes; dlqi_5 = social/leisure activities; dlqi_6 = sport; dlqi_7 = working/studying; dlqi_8 = interpersonal problems; dlqi_9 = sexual difficulties; dlqi_10 = treatment difficulties. *Response options are disordered. *HRQoL* health related quality of life *NRR* ‘not relevant’ response

In case of zero-scoring NRRs, item locations ranged from -0.27 to 1.94 logits, where item 1 (itchy/sore/painful/stinging) and 9 (sexual difficulties) were the least and most difficult (i.e. required the least and the most HRQoL impairment to endorse the item). Overall, examination of the person-item map suggests adequate coverage of items around the middle range of latent trait, but regardless of how NRRs were interpreted, a large proportion of patients had a high probability for low scores (Fig. 4). With zero-scoring NRRs, the locations of items 6 (sport), 7 (working/studying) and 8 (interpersonal problems) were very close to each other (0.736, 0.793 and 0.743) suggesting an item redundancy. With missing-scoring, location of item 3 (shopping/home/gardening) was very close to that of item 9 (sexual difficulties) (1.110 vs. 1.095), and location of item 4 (clothing) to that of item 10 (treatment difficulties) (-0.038 vs. 0.001). The order of difficulty of the 10 items was similar with the two scoring methods. The four most difficult items were 9 (sexual difficulties), 3 (shopping/home/gardening), 7 (working/studying) and 8 (interpersonal problems) with zero-scoring NRRs, whereas 3 (shopping/home/gardening), 9 (sexual difficulties), 8 (interpersonal problems) and 7 (working/studying) when missing-scoring NRRs.

### Item invariance

With zero-scoring NRRs, six and three items showed DIF for gender and age, respectively (Table 2). A uniform DIF was found in the majority of instances. Items 3 (shopping/home/gardening) and 6 (sport) were the only items to demonstrate DIF for both demographic variables. Items 7 (working/studying), 8 (interpersonal problems) and 10 (treatment difficulties) were free from DIF. With missing-scoring NRRs, four and three items indicated DIF for gender and age, respectively. Items 5 (social/leisure activities), 7 (working/studying), 8 (interpersonal problems) and 10 (treatment difficulties) were free from DIF.

## Discussion

Rasch models have previously been applied to investigate the psychometrics of the DLQI in various patient populations, including psoriasis, atopic dermatitis, hand eczema, neurodermatitis and chronic arsenic exposure [15–21, 38]. Yet this is the first study to evaluate measurement functioning of the DLQI using a Rasch model considering both possible interpretations of NRRs.

Our most important finding is that psychometric properties of DLQI can largely vary depending on how NRRs are interpreted. While aspects related to patients, such as person fit and person separation reliability indices were similar when zero-scoring and missing-scoring NRRs, substantial differences were seen at item level. These differences are visible in terms of item locations, item fit, response scale and invariance. The items with the highest proportion of NRRs [6 (sport), 7 (working/studying) and 9 (sexual difficulties)] performed particularly weakly in the Rasch model. These items suffered from item misfit and item-level disordering. It seems that NRRs are responsible for the majority of item misfit and disordering. Treating NRRs as missing accommodated the disordering of response categories and also increased the difference between them. This interpretation of NRRs has also reduced the DIF observed.

Results of previous attempts to investigate the psychometrics of the DLQI using a Rasch model somewhat differ from our findings. The person separation reliability values (0.910–0.914) in this study were modestly higher than those from former studies (range: 0.82–0.88) [17, 19]. Twiss et al*.* reported that items 2 (embarrassed/self conscious) and 7 (working/studying) misfitted the model and items 6 (sport), 7 (working/studying), 8 (interpersonal problems) and 9 (sexual difficulties) indicated a disordered response threshold in psoriasis patients [19]. In their study, items 2 (embarrassed/self conscious), 4 (clothes), 6 (sport) and 8 (interpersonal problems) exhibited DIF by gender and items 5 (social/leisure activities) and 10 (treatment difficulties) by age. Another study with psoriasis patients by *Nijsten *et al*.* reported all DLQI items to display DIF by culture, but no items by age or gender [17]. In contrast, with zero-scoring NRRs, we detected the presence of DIF in items 2 (embarrassed/self conscious), 3 (shopping/home/gardening), 4 (clothes), 5 (social/leisure activities), 6 (sport) and 9 (sexual difficulties) by gender and in items 1 (itchy/sore/painful/stinging), 3 (shopping/home/gardening), and 6 (sport) by age. No matter how NRRs are interpreted, our findings confirm that the DLQI performs poorly in terms of establishing measurement invariance across subgroups of patients. The lack of measurement equivalence highlights that there may be important differences in how certain groups (e.g. males vs. females or younger vs. older) tend to interpret most DLQI items, and therefore, the differences detected between known-groups of patients should be treated with caution.

Available data are controversial with regard to the dimensionality of DLQI: in accordance with our results some studies provided evidence supporting its unidimensionality [15, 21], while other studies revealed a multidimensional structure of the scale [16, 19, 20, 39]. Unidimensionality cannot be assessed without considering the content of the instrument [40, 41]. DLQI undoubtedly covers numerous aspects of HRQoL; even the developers suggest that the DLQI can be analysed under the following six subscales: symptoms and feelings (items 1 and 2), daily activities (items 3 and 4), leisure (items 5 and 6), work and school (item 7), personal relationships (items 8 and 9) and treatment (item 10) [2]. It may be argued that all these concepts underlie different constructs and a total score may not be calculated by summing the individual items. Thus, while our study confirmed the psychometrically unidimensional nature of the DLQI, subsequent studies are warranted to further investigate this finding.

Our findings have important implications for clinical practice and research. NRRs may lead to bias in the assessment of HRQoL and preclude meaningful comparisons across psoriasis patients. In numerous national guidelines on systemic treatment, psoriasis is considered moderate-to-severe if the patient has a PASI ≥ 10 and DLQI > 10, and a ≥ 5-point decrease in DLQI marks an adequate treatment response [42–44]. This latter is based on the average minimal clinically important difference of four points in inflammatory skin conditions [1, 45–48]. Given the central role of DLQI in these criteria, providing evidence of robust measurement properties is essential. It seems, however, that NRRs lead to measurement bias in the DLQI suggesting that scores of patients with at least one compared to no NRRs should not be compared. In the most extreme instance, by ignoring NRRs, one may risk underdiagnosis and even undertreatment of patients with moderate-to-severe psoriasis. Furthermore, consistently with previous Rasch models [15, 16, 19], the DLQI showed a limited ability to measure HRQoL in patients with very mild and extremely severe HRQoL impairment, since there are no items for patients with the worst and best HRQoL. The DLQI thus may need some more easy and difficult items (more and less likely endorsed, respectively), to achieve a better person-item targeting and be able to better discriminate between patients.

The better measurement performance of the DLQI with missing-scoring suggests that scoring NRRs equal to ‘not at all’ responses may be incorrect, as NRRs seem to represent a mixture of the other four response categories of the DLQI. A promising approach to resolve bias associated with NRRs may be an alternative scoring of the DLQI, the DLQI-R [12]. DLQI-R, in fact, missing-scores all NRRs and then replaces them with the average score of the relevant items. By this, the DLQI-R establishes a common ground to compare HRQoL of patients with at least one vs. no NRRs or of those ticking NRRs on different DLQI items. In the past three years, a growing number of observational studies reported the DLQI-R in psoriasis, pemphigus, morphea, vitiligo and hidradenitis suppurativa patients [46, 49–53]. Additionally, it is used as a primary endpoint in ongoing clinical studies with an interleukin-23 blocker, tildrakizumab for the treatment of psoriasis [54, 55]. Although the DLQI-R is equally good or even better in many aspects of measurement properties than the DLQI [12, 52, 53], it cannot settle all issues around the underlying construct of the original questionnaire (e.g. DIF).

One of the strengths of this study is that it included a heterogeneous study population that covered the full spectrum of psoriasis patients of varying age, gender, type of psoriasis and disease severity. We used a Rasch model analysis that represent a state-of-the-art technique for questionnaire development and validation [14]. Limitations of this study include the following. Referral bias cannot be ruled out, as both cross-sectional surveys were carried out at two academic dermatology clinics where more severe patients are referred to. Selection bias may also arise, since nearly half of the patients were treated with biologics as a preset inclusion criteria in one of the cross-sectional surveys [22]. This study used the Hungarian version of the DLQI, and thus, caution is warranted in generalising the results to other diagnoses and cultures. Lastly, a partial credit model was employed in the present study; nevertheless, other modelling approaches may be more suitable for other samples.

In conclusion, measurement performance of the DLQI varies depending on how NRRs are interpreted. It seems that NRRs should be treated as missing responses that significantly improve measurement properties, including item fit, response ordering and measurement invariance. These findings give further empirical support for the use of the DLQI-R scoring modification. Further research efforts need to be directed towards making effective revisions of the DLQI as well as standardizing HRQoL measurement in dermatology.

## Data Availability

All data of this study are available from the corresponding author upon reasonable request.
